# Supplementary material for: Seminoma and Embryonal Carcinoma Footprints Identified by Analysis of Integrated Genome-Wide Epigenetic and Expression Profiles of Germ Cell Cancer Cell Lines
Source: PLoS One. 2014 Jun 2;9(6):e98330. doi: 10.1371/journal.pone.0098330 (PMC4041891; doi:10.1371/journal.pone.0098330)
Supplement: File S1 — ZIP file containing DMRforPairs output for significant regions. Please start from the html files. (ZIP) [file pone.0098330.s008.zip › figures/27149.pdf]

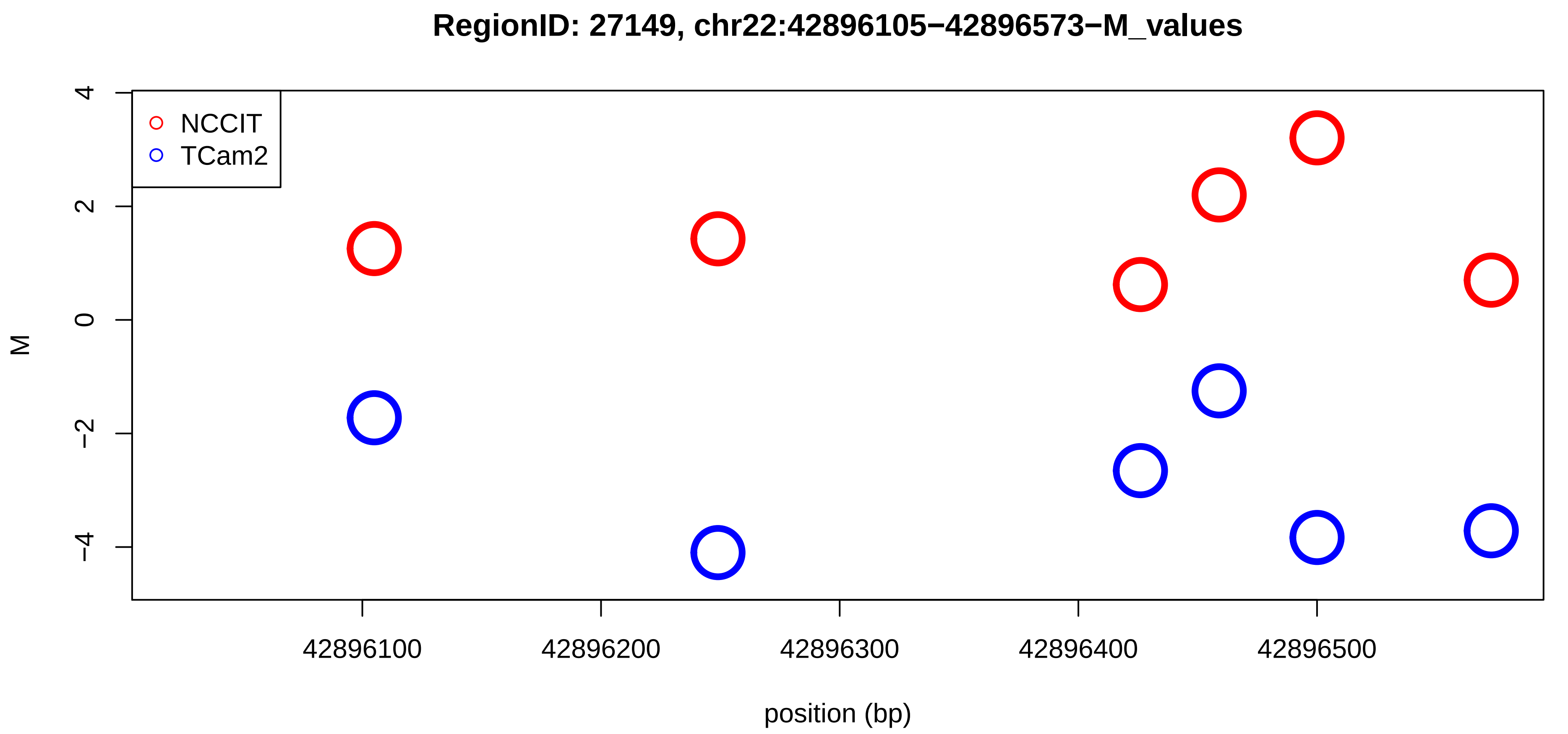

RegionID: 27149, chr22:42896105−42896573−M_values
M
NCCIT
TCam2
4
2
0
−2
−4
42896100
42896200
42896300
42896400
42896500
position (bp)

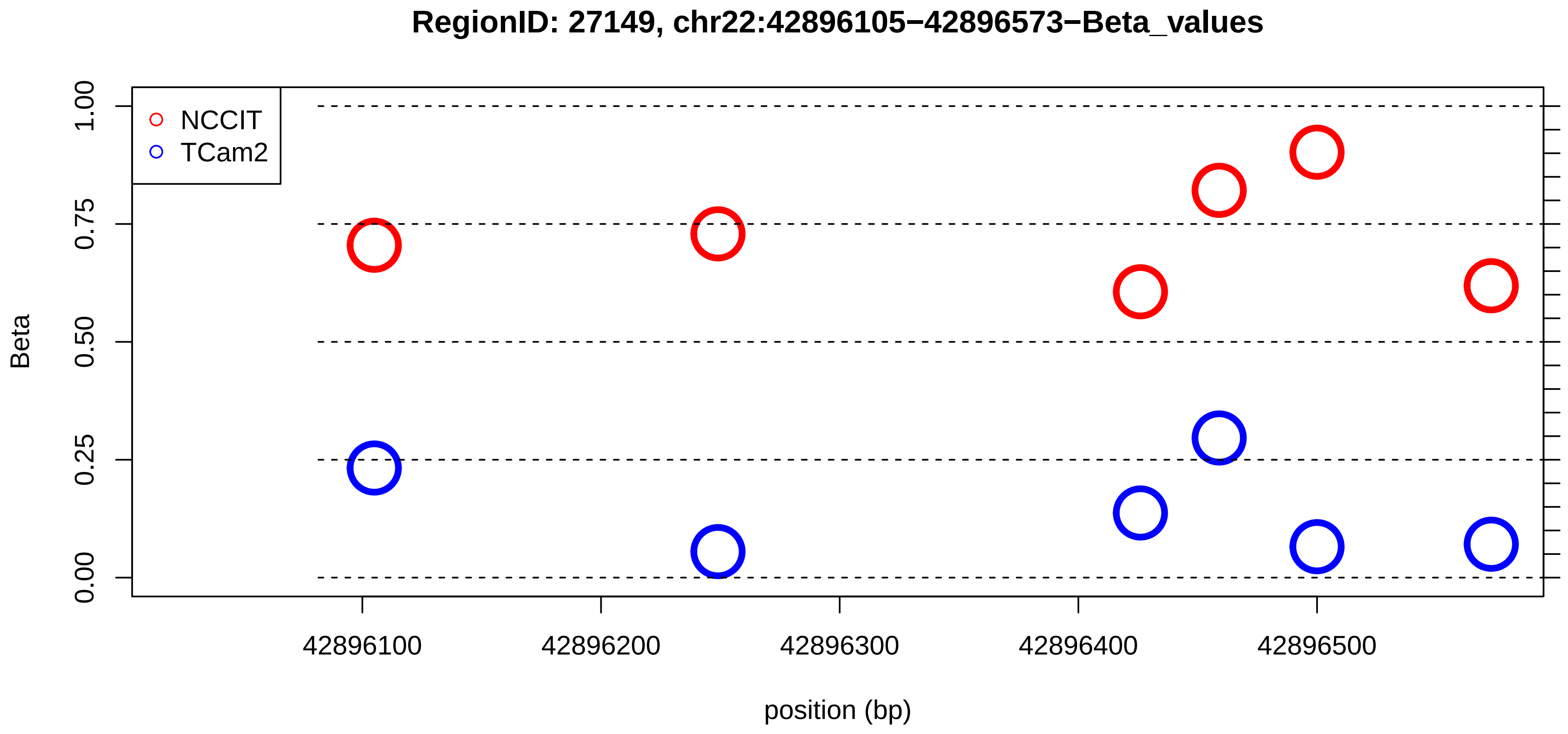

RegionID: 27149, chr22:42896105−42896573−Beta_values
Beta
NCCIT
TCam2
1.00
0.75
0.50
0.25
0.00
42896100
42896200
42896300
42896400
42896500
position (bp)
